# Clinical utility of *TGFB1* and its receptors (*TGFBR1* and *TGFBR2*) in thyroid nodules: evaluation based on single nucleotide polymorphisms and mRNA analysis

**DOI:** 10.20945/2359-3997000000330

**Published:** 2021-02-25

**Authors:** Karina Colombera Peres, Larissa Teodoro, Laís Helena Pereira Amaral, Elisângela Souza Teixeira, Icléia Siqueira Barreto, Leandro Luiz Lopes de Freitas, Valdemar Maximo, Lígia V. Montalli Assumpção, Natassia Elena Bufalo, Laura Sterian Ward

**Affiliations:** 1 Universidade Estadual de Campinas Faculdade de Ciências Médicas Laboratório de Genética Molecular do Câncer Campinas SP Brasil Laboratório de Genética Molecular do Câncer, Faculdade de Ciências Médicas, Universidade Estadual de Campinas, Campinas, SP, Brasil.; 2 Universidade Estadual de Campinas Faculdade de Ciências Médicas Departamento de Anatomia Patológica Campinas SP Brasil Departamento de Anatomia Patológica, Faculdade de Ciências Médicas, Universidade Estadual de Campinas, Campinas, SP, Brasil.; 3 Universidade do Porto Instituto de Investigação e Inovação em Saúde Porto Portugal Instituto de Investigação e Inovação em Saúde (i3S), Universidade do Porto, Porto, Portugal.; 4 Universidade do Porto Instituto de Patologia e Imunologia Molecular Porto Portugal Instituto de Patologia e Imunologia Molecular da Universidade do Porto (Ipatimup), Porto, Portugal.; 5 Universidade do Porto Faculdade de Medicina Departamento de Patologia Porto Portugal Departamento de Patologia, Faculdade de Medicina da Universidade do Porto (FMUP), Porto, Portugal.; 6 Universidade Estadual de Campinas Faculdade de Ciências Médicas Departamento de Medicina Campinas SP Brasil Divisão de Endocrinologia, Departamento de Medicina, Faculdade de Ciências Médicas, Universidade Estadual de Campinas, Campinas, SP, Brasil.

**Keywords:** Thyroid cancer, transforming growth factor-β, polymorphism, mRNA expression

## Abstract

**Objective:**

Abnormalities involving the *TGFB1* gene and its receptors are common in several types of cancer and often related to tumor progression. We investigated the role of single nucleotide polymorphisms (SNP) in the susceptibility to cancer, their impact on its features, as well as the role of mRNA expression of these genes in thyroid malignancy.

**Materials and methods:**

We genotyped *TGFB1*, *TGFBR1*, and *TGFBR2* SNPs in 157 papillary thyroid cancer (PTC) patients and 200 healthy controls. Further, we investigated RNA samples of 47 PTC and 80 benign nodules, searching for differential mRNA expression.

**Results:**

SNPs rs1800472 and rs1800469 were associated with characteristics of PTC aggressiveness. Effect predictor software analysis of nonsynonymous SNP rs1800472 indicated increasing protein stability and post-translational changes. *TGFB1* mRNA expression was upregulated in PTC and downregulated in benign samples, differentiating malignant from benign nodules (p<0.0001); PTC from goiter (p<0.0001); and PTC from FA (p<0.0001). *TGFBR1* mRNA expression was upregulated in goiter and PTC, but downregulated in FA, distinguishing PTC from goiter (p=0.0049); PTC from FA (p<0.0001); and goiter from FA (p=0.0267). On the other hand, *TGFBR2* was downregulated in all histological types analyzed and was not able to differentiate thyroid nodules.

**Conclusion:**

*TGFB1* polymorphism rs1800472 may confer greater activity to TGF-β1 in the tumor microenvironment, favoring PTC aggressiveness. Evaluation of *TGFB1* and *TGFBR1* mRNA levels may be useful to identify malignancy in thyroid nodules.

## INTRODUCTION

The Brazilian National Institute of Cancer (INCA) estimates about 12,000 new cases of differentiated thyroid cancer (DTC) for 2020, placing it as the fifth most incident cancer in women (1). Although most guidelines restrict the indication for further investigation of small nodules, and the criteria for malignancy have been more and more rigorous, an increasing number of patients end up referred to fine-needle aspiration (FNA) biopsy for diagnostic confirmation and many are submitted to surgery. It is fundamental to find ways to optimize the management of these patients, avoiding inappropriate and excessive spending on the health system, besides ensuring patients’ physical and psychological well-being (2).

Transforming growth factor-β1 (TGF-β1) is a multifunctional cytokine that plays a role in critical functions such as cellular differentiation, migration, apoptosis, and regulation of the immune systems (3). Simply, in epithelial cells, TGF-β1 signaling occurs by its binding with transforming growth factor-β receptor II (TβRII), which in turn recruits and phosphorylates transforming growth factor-β receptor I (TβRI), forming a heterodimeric complex. Once the type I receptor is phosphorylated, it can downstream phosphorylate proteins SMAD2 and SMAD3, which then recruits SMAD4 and now can translocate to the nucleus and regulate the transcription of TGF-β1 target genes (4). TGF-β1 plays an important role in the inhibition of thyroid cell proliferation and the modulation of the extracellular matrix. Cancer cells can explore processes modulated by TGF-β1, such as cell invasion and microenvironment modification, for their advantage. In the presence of an aberration of its normal signaling, the multifunctional role of TGF-β1 makes several pathological disturbances susceptible (5,6). Both mRNA and protein expression of TGF-β1 have been extensively investigated in a series of human cancers, including thyroid cancer; however, the potential of TGF-β1 as a clinical tool for the diagnosis and prognosis of thyroid tumors has not been thoroughly investigated. Besides, the literature still lacks reports describing the possible clinical utility of the expression of TGF-β1 receptors in thyroid cells.

Single nucleotide polymorphisms (SNP) are genetic variations often distributed throughout the human genome, and their location can interfere in different biological processes (reviewed in 7). Easily accessed nowadays, these SNPs can provide valuable information by identifying individuals genetically susceptible to multifactorial diseases, the aggressiveness of the disease, and poor response to treatments.

To better understand their role in the susceptibility and clinical features of thyroid cancer, we analyzed some *TGFB1*, *TGFBR1*, and *TGFBR2* SNPs previously associated with human cancers as well as SNPs that have been implicated on gene and protein deregulation (8–11). Intronic SNPs such as *TGFB1* rs8110090, rs2241716, rs11466321, rs1800469 and *TGFBR1* rs10512263, can lead to deregulation of gene expression: besides affecting the process of splicing, intron regions contain microRNA (miRNA) genes whose structure, processing, and function could be affected by nucleotide changes. SNPs at 5’ and 3’ untranslated region (UTR) are capable to affect mRNA translation and stability, respectively. *TGFBR1* rs7850895 was selected by its location in 3’ UTR where damages can impair mRNA-miRNA interaction. The SNPs *TGFB1* rs1800472 and *TGFBR2* rs2228048 are located in coding sequences and can affect protein structure, function and/or activity. Furthermore, *TGFB1* rs1800472 was previously associated with decreased risk to thyroid nodules (12). Next, based on in silico analysis of the possible impact of a nonsynonymous SNP (nsSNP), we investigated mRNA expression of *TGFB1* and its receptors in a well-characterized group of thyroid nodule patients carefully followed-up by a same group of health-care providers for a relatively long time.

## MATERIALS AND METHODS

### Subjects

The Research Ethics Committees of our institution approved this retrospective study (CAAE 38333014.2.0000.5404 and 53581416.3.0000.5404). We evaluated a total of 237 thyroid nodule patients submitted to partial or total thyroidectomy (194 women and 43 men, 43.8 ± 13.6 years old) consecutively referred to the Thyroid Cancer Unit, Division of Endocrinology, University of Campinas Teaching Hospital in Campinas, São Paulo, Brazil. There were 80 benign nodules (54 goiters and 26 follicular adenomas [FA]) and 157 papillary thyroid carcinomas (PTC) – 144 classic PTC (CPTC) and 13 follicular variant of PTC (FVPTC). Also, 14 normal thyroid (NT) tissue samples were obtained from the contralateral lobe of patients with benign FA for technique calibration purposes. FVPTC suspected of noninvasive follicular thyroid neoplasm with papillary-like nuclear features (NIFTP) were excluded from this study. These 237 samples derived from 127 formalin-fixed paraffin-embedded (FFPE) tissues (all 80 benign and 47 PTC) and the remaining PTC (110) from blood samples. DNA and/or RNA were extracted as detailed below.

Table 1 summarizes the clinical and anatomopathological characteristics of PTC patients. Individual sociodemographic characteristics and nodule characteristics, such as concurrent lymphocytic thyroiditis (CLT), multifocality, encapsulation, extra-thyroidal extension (EE), invasion and metastasis at diagnosis, were obtained from the patients’ charts and confirmed by two pathologists (ISB, LLLF). Thyroid cancer patients were monitored using serum TSH and thyroglobulin measurements, periodic cervical ultrasonography, and other eventual methods according to a standard protocol based on the American Thyroid Association (13) and Latin American Thyroid Association (14) recommendations. They were followed-up for 8.2 ± 3.3 years. Patients with thyroid cancer were classified as disease-free when they maintained unstimulated serum Tg levels <2 ng/dL and exhibited no clinical or image suspicion of disease for at least 12 consecutive months after surgery. Patients with anatomical evidence of metastasis were classified as recurrent (02 patients) and patients with persistent unstimulated serum Tg Levels >2ng/dL or with increasing Tg or Tg antibody serum levels were considered biochemically not-cured or undetermined (01 patient).

**Table 1:** Percentage of clinical and anatomopathological characteristics of 157 PTC patients, subdivided into classic PTC (CPTC) and follicular variant of PTC (FVPTC)

Characteristics				CPTC n = 144	FVPTC n = 13
X±SD	Tumor size (cm)			1.4 ± 1.0	2.2 ± 1.4
	Age at diagnosis			41 ± 12	51 ± 18
%	Sex		Women	78.5	92.3
			Men	21.5	7.7
		Presence of	Multifocality	27.7	46.2
			CLT	19.4	7.7
			Capsule	33.3	30.8
			Invasion	31.3	23.0
			LNM at diagnosis	22.9	15.4
	Outcome		Disease-free	97.9	100.0
			Undetermined	0.7	0.0
			Recurrent	1.4	0.0

Note: LNM, lymph node metastasis.

In addition, 200 blood samples were obtained from healthy blood donors (158 women and 42 men, 42.6 ± 11.3 years old) recruited at the Center of Hematology and Hemotherapy of the University of Campinas, Brazil. None of these control individuals had any history of thyroid disease.

### Genotyping

We genotyped a total of 157 PTC and 200 healthy individuals using TaqMan SNP genotyping assays (Applied Biosystems, CA, USA) with 7500 Real-Time PCR System (Applied Biosystems, CA, USA). A total of 110 DNA samples were extracted from blood by a standard protocol using phenol-chloroform and 47 from FFPE tissues using RecoverAll™ Total Nucleic Acid Isolation Kit (Life Technologies Corporation, California, USA), according to the manufacturer instructions. DNA samples were quantified, diluted to a final concentration of 20ng/µl, and genotyped for *TGFB1*, *TGFBR1* and *TGFBR2* SNPs detailed in Table 2.

**Table 2:** Description of the selected *TGFB1*, *TGFBR1* and *TGFBR2* SNP's

Gene	rs	Location	Region	Nucleotide exchange*	Amino acid exchange
*TGFB1*	rs8110090	Chr.19: 41339967	Intron	[A/G]	–
	rs2241716	Chr.19: 41348181	Intron	[C/T]	–
	rs11466321	Chr.19: 41349011	Intron	[A/G]	–
	rs1800472	Chr.19:41341955	Exon 5	[G/A]	Thr263Ile
	rs1800469	Chr.19:41354391	Intron	[A/G]	–
*TGFBR1*	rs7850895	Chr.9: 99153794	UTR 3	[T/C]	–
	rs10512263	Chr.9: 99123789	Intron	[T/C]	–
*TGFBR2*	rs2228048	Chr.3: 30672350	Exon 3	[C/T]	Asn389Asn

Note: Chr, chromosome; UTR3, untranslated region 3; Thr, Threonine; Ile, Isoleucine, Asn, Asparagine.

* According to dbSNP database (NCBI).

### In silico analysis

An effect predictor software was used to evaluate nsSNP. Information on the only nsSNP rs1800472 was obtained from the NCBI dbSNP database (https://www.ncbi.nlm.nih.gov/projects/SNP/), and the amino acid sequence of the protein was obtained from the Uniprot database (https://www.uniprot.org/).

PredictSNP1.0 (15) was used to evaluate the effect of the amino acid change on protein structure and function. This bioinformatic resource is a consensus classifier that allows access to performing prediction tools [SIFT (Sorting Intolerant from Tolerant), PolyPhen-1, PolyPhen-2, MAPP (Multivariate Analysis of Protein Polymorphism), PhD-SNP (Predictor of human Deleterious Single Nucleotide Polymorphisms), SNAP (Screening for Non-Acceptable Polymorphisms), PANTHER (Protein Analysis Through Evolutionary Relationships), PredictSNP, and nsSNPAnalyzer] and displays a consensus prediction by confidence scores observed in each tool. Also, we analyzed the evaluated SNP using three complementary tools: Align GVGD (16), which combines the biophysical characteristics of amino acids and protein multiple sequence alignments; MuPRO (17), for predicting protein stability changes; and ModPred (18), for predicting potential post-translational modifications.

### mRNA quantification

One hundred and forty-one RNA samples (54 goiters, 26 FA, 43 CPTC, 4 FVPTC, 14 NT) were randomly chosen and extracted from FFPE tissues using RecoverAll™ Total Nucleic Acid Isolation Kit (Life Technologies Corporation, California, USA). RNA samples were submitted to reverse transcription technique using the High-Capacity cDNA Reverse Transcription kit (Applied Biosystems™), also according to the manufacturer instructions. Afterward, qPCR assays were performed using inventoried TaqMan Gene Expression probes for *TGFB1* (Hs00998133_m1), *TGFBR1* (Hs00610320_m1), *TGFBR2* (Hs00234253_m1), and GAPDH (Hs02758991_g1) with 7500 Real-Time PCR System (Applied Biosystems, CA, USA). We used the 2^−ΔΔCT^ method (19), in which fold change is obtained by target gene expression normalized to an endogenous reference gene (GAPDH) and relative to 14 normal thyroid tissue.

### Statistical analysis

The statistical analysis was carried out using SAS System (Statistical Analysis System) for Windows, version 9.4 (SAS Institute Inc, 2002-2008, Cary, NC, USA), and graphs were drawn in GraphPad Prism 6 (GraphPad Software, Inc.). Haploview (20) was used to calculate the Hardy-Weinberg Equilibrium (HWE) and Linkage Disequilibrium between SNPs. Chi-square or Fisher's exact tests were used to study homogeneity between cases and controls. Non-parametric tests (Mann-Whitney or Kruskal-Wallis) were used to compare continuous or arranged measures between the groups. Data were expressed as median and interquartile range. The accuracy of gene expression studies to predict malignancy was evaluated using a receiver operating curve (ROC) analysis based on predicted probabilities from logistic regression models. P-value was two-sided and p<0.05 was considered statically significant.

## RESULTS

### Genotyping and haplotypes

The genotype distribution of *TGFB1*, *TGFBR1*, and *TGFBR2* polymorphisms for 157 PTC patients and 200 controls are shown in Table 3. All polymorphisms analyzed were in Hardy-Weinberg equilibrium (p>0.05). Polymorphic genotypes of rs8110090 *TGFB1* were more frequent in control individuals than in patients (p=0.0438), whereas the heterozygous variant CT of rs2228048 *TGFBR2* was numerically more frequent in PTC patients, although there was no statistically significant difference between cases and controls (p=0.0459). None of the polymorphisms was associated with PTC histological type. Patients with the heterozygous genotype (AG) of rs1800472 polymorphism presented a higher frequency of lymph node metastasis (LNM) at diagnosis compared with wild type patients (OR=3.625, 95%CI: 1.124-11.690, p=0.0433). Patients carrying polymorphic genotypes of rs1800469 polymorphism had a greater chance of having not encapsulated thyroid tumors (OR=3.109, 95%CI: 1.307-7.396, p=0.0105). The remaining SNPs and clinical feature comparisons are described in the Online Resource (Online Resource 1 and 2).

**Table 3:** Number and percentage of allele and genotype distribution of *TGFB1, TGFBR1* and *TGFBR2* genes in 157 PTC and 200 control individuals

Gene	PTC n (%)	Control n (%)	P-value	MAF Case-Control	HWEp
*TGFB1 rs8110090*					
A*	294 (94)	361 (90)	0.1315^a^	0.083	0.8766
G	20 (06)	39 (10)			
AA*	139 (89)	162 (81)	0.0703^b^		
AG	16 (10)	37 (18)			
GG	02 (01)	01 (01)			
AA vs AG+GG	18 (11)	38 (19)	**0.0438** c		
*TGFB1 rs2241716*					
C*	306 (97)	388 (96)	0.5199^a^	0.029	0.5226
T	08 (03)	14 (04)			
CC*	149 (95)	188 (93)	–		
CT	08 (05)	12 (06)			
TT	0 (0)	01 (01)			
CC vs CT+TT	08 (05)	13 (07)	0.8186^c^		
*TGFB1 rs11466321*					
A*	299 (95)	374 (94)	0.4181^a^	0.057	0.1978
G	15 (05)	26 (06)			
AA*	143 (91)	176 (88)	0.6392^b^		
AG	13 (08)	22 (11)			
GG	01 (01)	02 (01)			
AA vs AG+GG	14 (09)	24 (12)	0.3187^c^		
*TGFB1 rs1800472*					
G*	301 (96)	392 (98)	0.1181^a^	0.029	1.0
A	13 (04)	08 (02)			
GG*	144 (92)	192 (96)	–		
AG	13 (08)	08 (04)			
AA	0 (0)	0 (0)			
GG vs AG+AA	13 (08)	08 (04)	0.0970^c^		
*TGFB1 rs1800469*					
A*	196 (62)	240 (60)	0.5366^a^	0.388	0.9814
G	118 (38)	160 (40)			
AA*	63 (40)	69 (35)	0.4628^b^		
AG	70 (45)	102 (51)			
GG	24 (15)	29 (14)			
AA vs AG+GG	94 (60)	131 (65)	0.3203^c^		
*TGFBR1 rs7850895*					
T*	292 (93)	366 (92)	0.4864^a^	0.079	0.1896
C	22 (07)	34 (08)			
TT*	135 (86)	166 (83)	–		
CT	22 (14)	34 (17)			
CC	0 (0)	0 (0)			
TT vs CT+CC	22 (14)	34 (17)	0.5139^c^		
*TGFBR1 rs10512263*					
T*	293 (93)	368 (92)	0.5664^a^	0.075	0.2355
C	21 (07)	32 (08)			
TT*	137 (87)	171 (85)	0.7144^b^		
CT	19 (12)	26 (13)			
CC	01 (01)	03 (02)			
TT vs CT+CC	20 (13)	29 (15)	0.7569^c^		
TGFBR2 rs2228048					
C*	307 (98)	398 (99)	**0.0478** a	0.013	1.0
T	07 (02)	02 (01)			
CC*	150 (96)	198 (99)	–		
CT	07 (04)	02 (01)			
TT	0 (0)	0 (0)			
CC vs CT+TT	07 (04)	02 (01)	**0.0466** c		

Note:

* Reference allele and genotype

a Fisher's exact test

b Chi-square test

c Fisher's exact test – reference genotype *versus* variants genotypes;

MAF: minor allele frequency; HWEp: Hardy-Weinberg equilibrium p-value.

*TGFB1* polymorphisms were in linkage disequilibrium, and haplotype analysis was performed, as shown in Table 4. Seven haplotypes were generated for five selected SNPs (rs8110090, rs2241716, rs11466321, rs1800472, and rs1800469). The most frequent haplotypes in thyroid cases were AGCGG (48%) and AGCGA (32%). None of the haplotypes was associated with significant risk for PTC.

**Table 4:** Distribution of haplotype analysis of *TGFB1* SNPs (rs8110090, rs2241716, rs11466321, rs1800472, and rs1800469 in 157 PTC and 200 control individuals

Haplotype Associations	Frequency	Case ratio	Control ratio	p-value
AGCGG	0.486	0.522	0.457	0.0849
AGCGA	0.326	0.307	0.342	0.3266
AGCAG	0.053	0.043	0.061	0.2783
GGCGC	0.042	0.036	0.046	0.5141
GGCGA	0.034	0.025	0.041	0.2295

### In silico analysis

According to the conserved domains database of NCBI (https://www.ncbi.nlm.nih.gov/Structure/cdd/wrpsb.cgi), TGF-β1 has two well-conserved regions: TGF-beta pro-peptide domain from 29 to 261 amino acid position and *TGFB1* properly active from 293 to 390; rs1800472 is located on 263, therefore, in a non-conserved region of the *TGFB1* gene. Indeed, in silico analysis of rs1800472 by SIFT, which evaluated the impact of amino acid change based on the protein sequence homology in the evolutive process, showed similar results, classifying it as tolerated. Align GVGD predicts structural impact in the protein regarding biophysical characteristics of the amino acids, and this tool ranked T263I alteration as C65, indicating a negative impact in the protein structure. The remaining bioinformatic tools demonstrated no effective impact on the structure or function of TGF-β1 (neutral by PredictSNP, MAPP, PhD-SNP, PolyPhen-1, SNAP, PANTHER, and PROVEAN; not found by nsSNPAnalyzer). Furthermore, rs1800472 changes the protein by increasing stability, as shown in MuPRO, and it is related to post-translational modifications in phosphorylation sites (ModPred score=0.72).

### mRNA expression of TGFB1, TGFBR1, and TGFBR2

**Figure 1:**
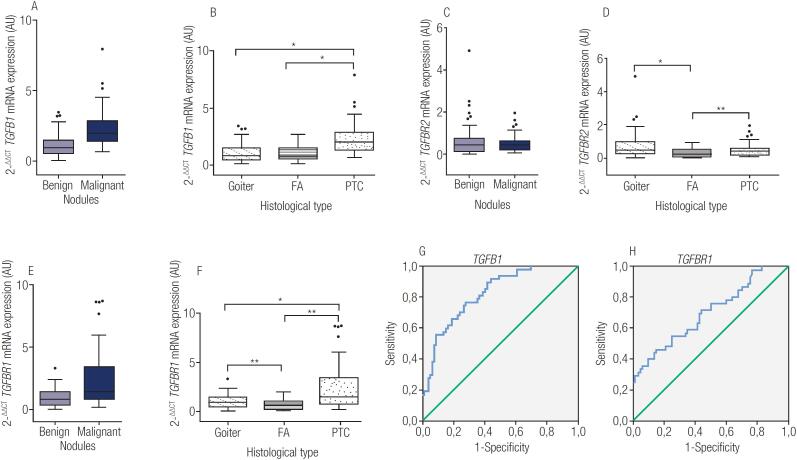
**A:** transforming growth factor-β1 (*TGFB1*) mRNA expression in 80 benign and 47 PTC, p<0.0001. **B:** *TGFB1* mRNA expression in the different types of tissues analyzed, *all comparisons p<0.0001. **C:** transforming growth factor receptor I (*TGFBR1*) mRNA expression in 80 benign and 47 PTC, p<0.0001. **D:** *TGFBR1* mRNA expression in the different types of tissues analyzed, *PTC versus goiter – p=0.0049, **PTC versus FA – p<0.0001, ***goiter versus FA – p=0.0267. **E:** transforming growth factor receptor II (*TGFBR2*) mRNA expression in 80 benign and 47 PTC, p-value not significant. **F:** *TGFBR2* mRNA expression in the different types of tissues analyzed, *goiter *versus* FA – p=0.0002, **PTC *versus* FA – p=0.0120. **G:** ROC analysis for *TGFB1* using a cut-off point of 1.365: sensitivity of 77%, specificity of 72%, PPV of 12%, NPV of 98%, and an area under the curve (AUC) of 0.821 (p<0.0001). **H:** ROC analysis for *TGFBR1* with sensitivity of 47%, specificity of 85%, PPV of 14%, NPV of 97%, and AUC of 0.701 (p=0.0002).

The expression of TGF-β1 and its receptors 1 and 2 mRNA was detected in all 127 samples (80 benign and 47 PTC). As shown in Figure 1A, mRNA expression of *TGFB1* was higher in malignant nodules compared to benign nodules (p<0.0001). A comparison among the histological types (Figure 1B) showed significant differences between PTC and goiter (p<0.0001) and between PTC and FA (p<0.0001).

Concerning receptor I of the *TGFB1* gene (*TGFBR1*), patients with malignant nodules also presented a higher mRNA expression than benign nodules (p<0.0001; Figure 1C). *TGFBR1* expression was able to distinguish PTC from goiter (p=0.0049), PTC from FA (p<0.0001), and goiter from FA (p=0.0267), as shown in Figure 1D. *TGFBR2* mRNA expression did not differentiate malignant from benign nodules (p=0.9732, Figure 1E), but distinguished goiter from FA (p=0.0002) and PTC from FA (p=0.0120), as shown in Figure 1F.

A binary logistic regression was performed to test the ability of *TGFB1* and *TGFBR1* mRNA expression to predict malignancy. A higher expression of these genes conferred to the patient with a nodule almost 4 (OR=3.553, 95%CI: 2.103-6.002, p<0.001) and 2 (OR=2.084, 95%CI: 1.396-3.112, p=0.0003) times more chances to have a malignant thyroid tumor, respectively.

A ROC curve analysis (shown in Figure 1G) suggested that *TGFB1* mRNA expression could distinguish malignant nodules with a sensitivity of 77%, specificity of 72%, positive predictive value (PPV) of 12%, and negative predictive value (NPV) of 98%, with an area under the curve (AUC) of 0.821 (cut-off 1.365 AU, p<0.0001). *TGFBR1* mRNA expression (Figure 1H), although presenting a significant p-value (p<0.0001), did not show a satisfactory AUC (0.701, sensitivity 47%, specificity 85%, PPV 14%, NPV 97%).

We were unable to demonstrate any association among clinical and pathological characteristics of the patients with *TGFB1*, *TGFBR1*, and *TGFBR2* mRNA expression (Table 5). Also, the low number of patients who evolved with metastasis (2 patients) or persistently elevated serum Tg levels (1 patient), precluded any further analysis on the impact of clinical and pathological characteristics and the investigated genes expression on patients’ outcome.

**Table 5:** mRNA expression of *TGFB1*, *TGFBR1*, and *TGFBR2* in 127 thyroid nodules and according to clinical/anatomopathological characteristics of 47 PTC patients

mRNA expression					TGFB1	TGFBR1	TGFBR2
**Nodules**	Normal tissue (n = 14)				0.99 ± 0.06	0.98 ± 0.07	0.99 ± 0.07
	Goiter (n = 54)				0.95 ± 0.61	1.09 ± 0.50	0.52 ± 0.65
	FA (n = 26)				0.83 ± 0.46	0.63 ± 0.40	0.21 ± 0.06
	PTC (n = 47)				1.95 ± 1.63	1.42 ± 1.65	0.42 ± 0.37
**Characteristics**	Sex			W	1.97 ± 1.50	1.31 ± 1.70	0.42 ± 0.37
				M	2.50 ± 1.90	1.62 ± 2.31	0.50 ± 0.46
	P-value				0.5164	0.3236	0.2676
	Multifocality			P	1.94 ± 1.40	1.50 ± 2.03	0.46 ± 0.28
				A	2.14 ± 1.67	1.14 ± 1.92	0.44 ± 0.43
	P-value				0.7320	0.6916	0.9272
	CLT		P		2.50 ± 1.80	0.91 ± 0.99	0.52 ± 0.76
			A		1.85 ± 1.45	1.53 ± 2.20	0.42 ± 0.32
	P-value				0.0637	0.1725	0.3931
	Capsule			P	1.49 ± 1.52	0.73 ± 1.38	0.29 ± 0.34
				A	2.10 ± 1.46	1.43 ± 1.91	0.47 ± 0.32
	P-value				0.3096	0.1409	0.1835
	Invasion	P			1.97 ± 1.57	1.42 ± 2.40	0.34 ± 0.30
		A			2.07 ± 1.78	1.20 ± 1.56	0.52 ± 0.31
	P-value				0.8063	0.4483	0.1690
	LNM			P	2.07 ± 1.16	1.56 ± 2.30	0.49 ± 0.58
				A	1.90 ± 1.64	1.08 ± 1.70	0.40 ± 0.35
	P-value				0.7320	0.3296	0.8640

Note: FA: follicular adenoma; PTC: papillary thyroid carcinoma; W: women; M: men; P: presence; A: absence; LNM: lymph node metastasis at diagnosis. Expression values expressed as arbitrary units (AU).

## DISCUSSION

First, in this study, we aimed to investigate the role of *TGFB1*, *TGFBR1*, and *TGFBR2* SNPs in the susceptibility to thyroid nodules malignancy and their correlation to clinical and anatomopathological characteristics. Although rs8110090 (*TGFB1*) and rs2228048 (*TGFBR2*) tended to be more frequently altered in controls and PTC, respectively, the relatively low number of individuals analyzed prevented the association of these SNPs to PTC susceptibility, since the data had a low power of calculation (48% and 33%, respectively). In addition, the relatively low number of FVPTC samples precluded further analysis of the PTC variants. In order to get a better sense of the putative clinical utility of *TGFB1* investigation in thyroid tissues, we further investigated mRNA expression of *TGFB1* and its receptors and tried to correlate genotype profile and mRNA expression. Unfortunately, due to the low MAF observed and the number of samples, we did not obtain significant results (Online resource 3).

We also observed that two polymorphisms of *TGFB1* were related to aggressiveness in PTC cases: both polymorphic genotypes of rs1800469 were frequent in patients with not encapsulated PTC and the heterozygous polymorphic genotype of rs1800472 was more frequent in patients with LNM at diagnosis. SNPs rs1800469 and rs1800472 have been vastly investigated in different types of cancer. Located in the negative regulatory region of the *TGFB1* gene, rs1800469 is associated with differential mRNA and plasma levels of TGF-β1 (reviewed in (8). Its association with cancer is still controversial once polymorphic and wild-type genotypes have been associated with susceptibility and/or aggressiveness (9,21,22). Considering rs1800472, our interest in this missense polymorphism (Thr263Ile) emerged from its location in a critical region for the activation of TGF-β1, which could affect conformation and function of the protein (reviewed in (8). This polymorphism was previously associated with decreased risk to thyroid nodules (OR=0.5 95%CI 0.3-0.8, p<0.0001) in a study with 879 patients, selected among a population living nearby Semipalatinsk nuclear test site, and 884 control individuals (12). However, in other case-control studies for bladder (23) and breast cancer (24), authors did not find any association of rs1800472 with susceptibility or prognosis. Here, we also performed a computational analysis seeking to predict how the amino acid change of rs1800472 could affect the protein's structure and function. Even though it was classified as tolerant or neutral for most of the in silico tools, two results caught our attention. First, the analysis by MuPRO (17) indicated that this polymorphism may result in increasing protein stability. In fact, a functional analysis performed by Thys and cols. (25) showed that luciferase activity of polymorphic 263Ile TGF-β1 variant was 21.2% higher than the wild-type variant (Thr263). Second, according to ModPred (18), this amino acid change is related to post-translational modification (PTM) in phosphorylation sites. Known, TGF-β1 is secreted in a latent form, binding with a latency-associated peptide (LAP), which prevents TGF-β1 signaling from being propagated to the nucleus; cleavage of LAP is critical for TGF-β1 activation. In fact, rs1800472 is a few amino acids away from the LAP cleavage point, thus, intuitively, this modification could be related to the loss of the phosphorylation site due to the exchange of threonine for isoleucine, being detrimental to the protein's activation. Nevertheless, this region also lacks amino acid sequence conservation, which is speculated to promote diversification in the TGF-β1 activation mechanism (26). TGF-β1 can be activated by a variety of molecules (e.g. proteases, metalloproteases, integrins, reactive oxygen species), most of them related to disturbance of the extracellular matrix (27). The tumor microenvironment (TME) is composed of extracellular matrix and other cellular components (endothelial cells and innate and adaptive immunity cells), making it a favorable environment for tumor development (27). TGF-β1 also promotes the expansion of Treg cells and the inhibition of effector T cells, antigen-presenting dendritic cells, and natural killer cells, as regulation of macrophages and neutrophils (28,29). TME is very heterogeneous among tumors and lesions from the same and different patients, even though the mechanisms responsible for this are poorly understood, genetic and epigenetic alterations may be involved (30,31). We suggest that, depending on the presence of rs180072 polymorphisms and the TME profile, TGF-β1 may have greater activity and affect PTC behavior. However, functional studies are needed to confirm this hypothesis. We did not observe any significant difference in mRNA expression and the corresponding genotypes, probably because of our relatively small sample size.

Furthermore, we analyzed the mRNA expression pattern of *TGFB1*, *TGFBR1*, and *TGFBR2* in malignant and benign thyroid tissues. We found that *TGFB1* mRNA expression was higher in PTC and lower in benign samples. These data corroborate previous reports. Kajdaniuk and cols. (32) were the first group to investigate *TGFB1*, *TGFBR1*, and *TGFBR2* mRNA expression simultaneously in thyroid tissues. The authors observed an elevated mRNA expression of *TGFB1* in PTC (n=06) compared to multinodular goiter (n=22, p=0.015) and Graves’ disease (n=08, p=0.001). In this same study, they performed a serum analysis of TGF-β1 that did not present differences (32), supporting the similar findings of Zivancevic-Simonovic and cols. (33) and suggesting a local pathological effect of the protein. Brace and cols. (34) also found an increased mRNA expression of *TGFB1* in 24 PTC compared to 23 goiters. Our data suggest *TGFB1* mRNA expression can help rule out malignancy in thyroid nodules with a NPV of 98% and deserves to be tested in FNA samples.

Our data showed that expression of *TGFBR1* was higher in goiter and PTC and lower in FA. Both the hyperplasia and tumorigenesis processes involve abnormal growth, eliciting increased mRNA expression of *TGFB1* and its receptor *TGFBR1*, the main driver of the TGF-β1 signaling cascade (4,35). On the other hand, *TGFBR2* was low in all histological types analyzed. Loss of *TGFBR2* expression in thyroid tumors was already reported in the 90's using Northern blot (36) and in-situ hybridization analysis (37). Matoba and cols. suggested that this decrease might lead the cell to escape from the negative inhibition of TGF-β1 (36).

Both receptors were also evaluated by Kajdaniuk and cols. (32), who did not find a difference for *TGFBR1*, but observed lower *TGFBR2* mRNA expression in all tissues analyzed, especially in PTC. RNA sequencing expression data extracted from GEPIA (38) also showed higher levels of *TGFBR1* and loss of *TGFBR2* expression (log2 fold change 1.349 AU and −1.738 AU, respectively) in 512 malignant thyroid tissues compared to 337 NT. Significantly higher *TGFBR1* mRNA levels were found in breast cancer patients with poor prognosis and small tumors as loss of *TGFBR2* mRNA was evidenced in primary breast tumors, but, curiously, higher levels of this gene were associated with better prognosis (39), which, added to in vivo and in vitro esophageal squamous cell carcinoma experiments, suggested that *TGFBR2* overexpression induces cell cycle arrest and suppress cell growth (40). Furthermore, recent research in cancer cell lines suggested that some miRNAs, such as miR-133b and miR-20b-5p, can inhibit the epithelial-mesenchymal transition (EMT) induced by TGF-β1 by targeting, respectively, *TGFBR1* and *TGFBR2* genes (41,42). As elucidated by Fuziwara and cols. in a recent review, a series of different microRNAs can target mRNA related with the TGF-β1 signaling pathway, and its deregulation is frequently seen in thyroid neoplasia (43).

In conclusion, our data suggest that some polymorphisms, such as rs1800472, may modulate TGF-β1 activity and help define PTC aggressiveness. In addition, evaluating *TGFB1* and *TGFBR1* mRNA levels may be useful to characterize thyroid nodules malignancy.

## Appendix

**Online resource 1:** Percentage of genotype distribution of *TGFB1* polymorphisms in the clinical/anatomopathological characteristics of 157 PTC patients

Characteristics			rs8110090		p-valu0	rs2241716		p-value	rs11466321		p-value	rs1800472		p-value	rs1800469			p-value*
			AA	AG+GG		CC	CT+TT		AA	AG+GG		GG	AG		AA	AG	GG	
**X´ ±SD**	Tumor size (cm)		1.7 ± 1.2	1.8 ± 1.4	0.8367	1.7 ± 1.3	1.6 ± 1.2	0.7513	1.7 ± 1.3	1.7 ± 1.0	0.3579	1.7 ± 1.3	1.9 ± 1.1	0.1030	1.8 ± 1.3	1.7 ± 1.2	1.3 ± 1.3	0.0531
	Age at diagnosis		44 ± 13	43 ± 13	0.8269	44 ± 13	44 ± 11	0.9797	44 ± 13	41 ± 11	0.2628	43 ± 13	47 ± 14	0.2804	43 ± 13	44 ± 13	43 ± 13	0.9115
**%**	Sex	W	88.0	12.0	1.0000	95.2	4.8	0.6659	92.0	8.0	0.4862	92.8	7.2	0.3036	37.1	48.4	14.5	0.1519
		M	90.6	9.4		93.8	6.3		87.5	12.5		87.5	12.5		51.6	29.0	19.4	
	Multifocality	P	91.3	8.7	0.7767	93.5	6.5	0.3841	91.3	8.7	1.0000	87.0	13.0	0.1987	31.1	55.6	13.3	0.2686
		A	87.8	12.2		97.0	3.0		90.8	9.2		94.0	6.0		43.3	41.2	15.5	
	CLT	P	89.8	10.2	1.0000	95.9	4.1	1.0000	85.7	14.3	0.2421	95.9	4.1	0.2027	42.3	43.6	14.1	0.7160
		A	88.6	11.4		96.2	3.8		92.4	7.6		88.6	11.4		36.7	51.0	12.2	
	Capsule	P	97.0	3.0	0.1748	93.8	6.2	0.3046	90.6	9.4	1.0000	89.3	10.7	0.7249	63.3	23.3	13.3	**0.0198**
		A	87.0	13.0		97.6	2.4		91.7	8.3		93.8	6.3		35.7	51.2	13.1	
	Invasion	P	91.7	8.3	0.4196	100.0	0.0	N.E.	85.4	14.6	0.1317	89.6	10.4	0.5424	46.2	38.5	15.4	0.3960
		A	86.0	14.0		92.5	7.5		93.5	6.5		92.5	7.5		35.4	50.0	14.6	
	LNM	P	91.4	8.6	0.3981	97.1	2.9	1.0000	85.7	14.3	0.1708	80.0	20.0	**0.0433**	45.7	45.7	8.6	0.5982
		A	84.9	15.1		96.8	3.2		93.5	6.5		93.5	6.5		40.2	44.6	15.2	

Note:

* p-value by Chi-square test. W: women; M: men; P: presence; A: absence; LNM: lymph node metastasis at diagnosis.

**Online resource 2:** Percentage of genotype distribution of *TGFBR1* and *TGFBR2* polymorphisms in the clinical/anatomopathological characteristics of 157 PTC patients

Characteristics			rs7850895		p-value	rs10512263		p-value	rs2228048		p-value
			TT	CT		TT	CT+CC		CC	CT	
**X´ ±SD**	Tumor size (cm)		1.7 ± 1.3	1.8 ± 1.4	0.8044	1.4 ± 1.1	1.5 ± 1.0	0.2993	1.4 ± 1.1	1.4 ± 0.5	0.5914
	Age at diagnosis		44 ± 13	43 ± 15	0.5041	42 ± 13	41 ± 15	0.3211	41 ± 12	53 ± 21	0.1886
**%**	Sex	W	85.6	14.4	1.0000	84.7	15.3	0.0897	95.2	4.8	1.0000
		M	87.1	12.9		96.8	3.2		96.8	3.2	
	Multifocality	P	84.4	15.6	0.8052	84.4	15.6	0.5877	95.6	4.4	1.0000
		A	85.7	14.3		88.8	11.2		96.0	4.0	
	CLT	P	83.7	16.3	0.7996	89.8	10.2	0.5932	95.9	4.1	1.0000
		A	86.1	13.9		84.8	15.2		94.9	5.1	
	Capsule	P	90.3	9.7	0.5519	83.9	16.1	0.7626	96.8	3.2	1.0000
		A	84.5	15.5		86.9	13.1		94.0	6.0	
	Invasion	P	83.3	16.7	0.4464	87.5	12.5	1.0000	97.9	2.1	0.6640
		A	88.0	12.0		85.9	14.1		94.6	5.4	
	LNM	P	85.7	14.3	1.0000	82.9	17.1	0.5751	97.1	2.9	1.0000
		A	86.0	14.0		87.0	13.0		94.6	5.4	

Note: W: women; M: men; P: presence; A: absence; LNM: lymph node metastasis at diagnosis.

**Online resource 3:** *TGFB1*, *TGFBR1* and *TGFBR2* polymorphisms and corresponding mRNA expression (median and interquartile range)

SNP (n)	mRNA expression	P-value
*rs8110090*	*TGFB1*	
AA (44)	2.03 ± 1.58	
AG (02)	2.17 ± 0.46	0.9162^a^
GG (01)	1.57	
*rs2241716*	*TGFB1*	
CC (46)	2.03 ± 1.49	
CT (01)	1.39	–
TT (00)	–	
*rs11466321*	*TGFB1*	
AA (43)	1.99 ± 1.44	
AG (04)	2.17 ± 5.48	0.7803^a^
GG (00)	–	
*rs1800472*	*TGFB1*	
GG (41)	1.99 ± 1.41	
AG (06)	1.92 ± 2.78	0.9846^a^
AA (00)	–	
*rs1800469*	*TGFB1*	
AA (16)	2.16 ± 1.55	0.8536^b^ 0.8718a*
AG (23)	1.95 ± 1.72	
GG (08)	1.98 ± 1.27	
*rs7850895*	TGFBR1	
TT (39)	1.42 ± 2.45	
CT (08)	1.86 ± 4.15	0.9221^a^
CC (00)	–	
rs10512263	*TGFBR1*	
TT (42)	1.56 ± 2.91	
CT (05)	0.94 ± 1.94	0.2892^a^
CC (00)	–	
*rs2228048*	*TGFBR2*	
CC (47)	0.42 ± 0.37	
CT (00)	–	–
TT (00)	–	

Note:

a Mann-Whitney test

a* AA versus AG+GG

b Kruskal-Wallis test.
